# *In vitro* complex formation of human PYRIN domain-only protein 3 prevented by self-oligomerization of ASC PYD domain

**DOI:** 10.1016/j.sjbs.2020.12.049

**Published:** 2021-01-05

**Authors:** Eijaz Ahmed Bhat, Nasreena Sajjad, Javeed Ahmad Tantray, Yan-Yan Hor, Irfan A. Rather

**Affiliations:** a Life Science Institute, Zhejiang University, Hangzhou, Zhejiang 310058, PR China; b Department of Biochemistry, University of Kashmir, Srinagar, 190006, India; c Department of Zoology, Central University of Kashmir, Ganderbal 191201, India; d Department of Biotechnology, College of Life and Applied Sciences, Yeungnam University, Gyeongsan 38541, South Korea; e Department of Biological Sciences, Faculty of Science, King Abdulaziz University, Jeddah 21589, Saudi Arabia; f Center of Excellence for Bionanoscience Research, King Abdulaziz University, Jeddah 21589, Saudi Arabia

**Keywords:** Innate immunity, POP3, ASC, Inflammasome, PYD domain

## Abstract

The formation of inflammasome complexes contributes inactivation of inflammatory caspases *viz* caspase 1, which is generally considered essential for the innate response. Three proteins constituted this inflammasome complex, such as Nod-like receptors (NLRP or AIM2), ASC possessing caspase-recruiting domain, and caspase-1. The ASC proteins comprise two domains, the N-terminal PYD domain responsible for the interaction of various proteins, including PYD only protein 3 (POP3), and the CARD domain for association with other proteins. The PYRIN Domain-Only Protein POP3 negatively regulates responses to DNA virus infection by preventing the ALR inflammasome formation. POP3 directly interacts with ASC, therefore inhibiting ASC recruitment to AIM2-like receptors (ALRs). In the current study, we designed various constructs of the PYRIN Domain-Only Protein 3 (POP3) and ASC PYD domain to find the best-overexpressed construct for biochemical characterization as well as our complex studies. We cloned, purified, and characterized the PYD domain of pyrin only protein 3 and ASC PYD domain under physiological conditions. Our *in vitro* study clearly shows that the ASC PYD domain of corresponding amino acid 1–96 aa with ease self-oligomerization in physiological buffer conditions, and complex formation of POP3 PYD (1–83 aa) was inhibited by ASC PYD domain. Besides, we purified the PYD of POP3 protein in low and high salt conditions and different pH values for their biochemical characterization. Our results showed that POP3 formed a dimer under normal physiological conditions and was stable under normal buffer conditions; however, the purification in extremely low pH (pH5.0) conditions shows unstable behavior, the high salt conditions (500 mM NaCl) influence the protein aggregation. SDS PAGE arbitrated the homogeneity of the PYD domain of pyrin only protein 3 and ASC PYD domain of corresponding amino acids 1–83 and 1–96, respectively. Furthermore, our native PAGE shows the PYD domain of pyrin; only protein 3 did not form a complex with ASC PYD domain because of oligomerization mediated by the PYD domain.

## 1 Introduction

The inflammasome complex constituted of a large number of proteins is responsible for the inflammatory caspase activation, like caspase-1 and caspase-5, which in turn produced matured and fully functional cytokines (Franchi et al., 2009a, Franchi et al., 2009b, Schroder and Tschopp, 2010). NLR and caspase-1 are the three proteins that constituted inflammasome (Schroder and Tschopp, 2010, Bae and Park, 2011). The Death domain superfamily is included in four subfamilies, including the DED subfamily, the CARD subfamily, and the subfamily of the PYD (Park, 2011, Park, 2012, Kwon et al., 2012). NLRs possess PYD-domain such as NLP3, which senses various danger signals, including bacterial toxins (Broz and Denise, 2011, Franchi et al., 2009a, Franchi et al., 2009b). ASC acts as an adaptor molecule, containing the PYD & CARD domain at the N-terminus and C-terminus responsible for the protein interaction module. During inflammasome formation, the ASC PYD domain interacts with NALP3 and caspase −1 (Schroder & Tschopp, 2010).

The PYD-only proteins (POPs) form the inflammasome regulator family (Dorfleutner et al., 2015). POPs are expressed in many species, including humans, but lack in mice, which suggests the evolution of an intricate mechanism of inflammasome regulation in few organisms (Khare et al., 2014). IL-1b & IL-17 are pro cytokines produced in an inactive form that has to be processed by enzymes for the activation of proper immune responses is critical (Ghayur et al., 1997; Sutterwala et al., 2006). To date, four pyrin-only protein members (POP1, PO2, POP3, POP4) are identified as inflammation with innate immunity regulators because of their wide role during inflammasome assembly (Chu et al., 2015). While the pseudogene NLRP2P has recently recognized POP4, it has 80% identification with POP2 (Porter et al., 2014a, Porter et al., 2014b). POP1 discovered by Stehlik et al. (also known as PYD-containing protein 1) was initially recognized as ASC2 due to strong homology with the domain of ASC PYD. The gene is present on 16p12.1 locus of chromosome and with two exons interjected by an intron of 580 bp (Stehlik et al., 2003). POP1 is involved in the regulation of inflammation. It is mainly present in macrophages, granulocytes, and monocytes. POP1 is implicated in NF-κB activation inhibition via numerous stimuli such as IL-1β, TNFα, Bcl-10, and Nod1, in COS-7, HEK-293, and Hela cells. Similarly, POP2 (also known as PYDC2) is responsible for interacting with the PYD of ASC and several NLRPs containing the PYD domain (Ratsimandresy et al., 2017). Furthermore, it is involved in the prevention of NLR-mediated activation of NF-κB. It is encoded by a nucleotide of 294 and contains a large protein with 97 amino acids similar to NLRP2 and NLRP7 PYDs (78% similarity, 67% identity NLRP2 PYD) (Le and Harton, 2013). Numerous stimuli like PMA, LPS, or TNF-α, induces POP2 in human primary monocytes, which indicates its role in inflammation and host immunity. Also, POP2 is expressed in leukocytes of peripheral blood, human testes, and primary, monocytic cell lines (Felipe et al., 2007). POP3 is encoded within the NLRP2P pseudogene by an ORF. The protein produced through this ORF is expected to demonstrate the NF-κB inhibitory possessions of POP2; nevertheless, it dearth the capability to hinder inflammasome NLRP3 (Porter et al., 2014a, Porter et al., 2014b). POP3inhibitsALR inflammasome formation by interacting with the AIM2 and IFI16 (Schroder & Tschopp, 2010). Research has shown that silencing POP3 in human macrophages increases ALR inflammasome development caused by DNA and DNA viruses, and thus, the expression of IL-18-18 and IL-1β (Schroder & Tschopp, 2010). Studies have revealed that impairment of ALR inflammasome response occurs by POP3 expression (Schroder and Tschopp, 2010, Indramohan et al., 2018). Therefore, POP3 has a key role in ALR inflammasome controller in macrophages of animal and clinical trials. ALR inflammasomes are blocked by a novel inhibitor known as POP3 and control the DNA virus infection I host defense (Christian et al., 2014). Inflammasome and innate immunity are mediated by PYD domain are linked with several human diseases such as immune disorders, including cancer and aging; research in such areas is of great diverse biological interest (Franchi et al., 2009a, Franchi et al., 2009b). Bacterial flagellin and the T3SS could trigger the NAIP-NLRC4 inflammasome (Amer et al., 2006, Franchi et al., 2006, Miao et al., 2006, Miao et al., 2010, Kofoed and Vance, 2011, Zhao et al., 2011, Mariathasan et al., 2004). Conversely, NLRC4 is not a clear sensor, collaborating by upstream NAIP proteins that assess the NLRC4 inflammasome bacterial ligand (Ren et al., 2006, Rauch et al., 2016, Zhao et al., 2016). AIM2 consists of a PYD N-terminal and a HIN-200 domain C-terminal and identifies cytosolic dsDNA in a sequence Independent but a length-dependent way through HIN-200 (Albrecht et al., 2005, Jin et al., 2012, Matyszewski et al., 2018). Several other, but less well-characterized, PRRs, including NLRP2, 6, 7, 9b, 12, IFI16, and NLRC5, have also been involved in inflammasome formation (Minkiewicz et al., 2013, Matsuoka et al., 2019, Elinav et al., 2011, Hara et al., 2018, Khare et al., 2012, Zhu et al., 2017, Vladimer et al., 2012, Davis et al., 2011a, Davis et al., 2011b, Kerur et al., 2011). In response to intracellular LPS detection of human caspase-4 and 5 as well as mouse caspase-11 (Hagar et al., 2013, Kayagaki et al., 2011, Kayagaki et al., 2013, Casson et al., 2015, Schmid-Burgk et al., 2015, Sollberger et al., 2012, Vigano et al., 2015). In our present study, we have successfully cloned the Human PYD domain of Pyrin only protein 3 and ASC PYD domain for their biochemical characterization together with molecular complex formation *in vitro*. The Ni-affinity chromatography and size exclusion chromatography (SEC) was used for protein purification. The main peak of the PYD domain of POP3 in SEC was eluted at 16 mL, which suggests the formation of a dimer in solution, and was found homogenous as was judged by the SDS-PAGE, which was subsequently characterized by a MALS. Furthermore, our study shows that in extremely low pH (pH5.0) conditions and high salt conditions (500 mM NaCl) influence the protein behavior and aggregation. The Native PAGE shows that the ASC PYD domain's self-oligomerization inhibited the formed complex with PYD domain of pyrin only protein 3 *in vitro* consistent with a study done by Narayanan KB et al, shows that ASC PYD easily self-oligomerized to prevent the formation of a complex with NALP3 PYD *in vitro*. Finally, the purity and homogeneity of the PYD domain of POP3 and ASC PYD, together with complex, were investigated by SDS-PAGE.

## 2 Material and methods

### 2.1 POP3PYD and ASCPYD cloning, expression & purification in *E. coli*

The cDNA of Human full-length PYRIN Domain-Only Protein 3 and ASCPYD domain were used to perform the polymerase chain reaction (PCR). Afterward, PCR products were digested using restriction enzymes. The digestion of the plasmid pET24a was carried. The PYD domain (1–83) of Pyrin only protein 3 and ASC PYD (1–96) were sub-cloned in a C-terminally His –tag (His6) plasmid (pET24a) (Novagen). Following, the transformation of the plasmids into the BL21 cell (DE3) competent cells. The agar plates possessing Luria Bertani (LB) comprising the appropriate antibiotics (50ul/mL) were used to gently spread each clone on it, following the incubation of agar plates for 16hr at 37 °C. A micro-loop was used to inoculate single colonies from each construct into a 5 mL LB medium, incubated overnight in a 37 °C shaking incubator. The next day, 5 mL of overnight pre-inoculated culture was inoculated into 2000 mL of the flask, which contained 100 mL LB media, and was incubated for 4 hrs at 37 °C with shaking. Also, the routinely tested optical density (O.D) until it reached between 0.6 and 0.7 nm, by 0.25 mM IPTG treatment at 20 °C overnight following protein overexpression. By centrifugation at 10,000*g*, the bacteria expressing each protein were pelleted, resuspended, and then sonicated in 40 mL of buffer (lysis) with added to phenyl methane sulfonyl fluoride (PMSF). Subsequently, centrifugation was carried out of lysate. After the addition of a 70 mL buffer (Wash), the unbound proteins were eliminated. Finally, the buffer (elution) was used to elute the target proteins. Total of 2 mL protein samples being collected with equally divided in 4* 0.5 mL eluted fractions. The SD-PAGE was used to check the purity of the target protein. The protein fractions containing more than 90% homogenous PYD domain of Pyrin only protein 3 were combined. The sample was loaded to the column superdex 200 at the final purification stage, which was pre-equilibrated with a 20 mM Tris-HCl solution at pH8.0 and 150mMNaCl. For biochemical characterization, the SEC PYD domain protein fractions were pooled & then stored at 4 °C. SDS-PAGE was used to judge the homogeneity of the PYD domain of Pyrin only protein- 3.Cloning, protein expression, and purification were conducted as described previously (Kim, 2016, Bhat et al., 2018, Bhat et al., 2020).

### 2.2 MALSxxx

To determine the absolute molecular mass, the PYD domain of Pyrin only protein 3 (1–83 aa) was used by a technique known as MALS. The protein of interest was purified by two rapid steps, Nickel-AC and SEC. Only protein 3 corresponding amino acids 1–83 were obtained from the key peak fractions. The centrifugation (10,000*g*) was followed by removing the precipitate at 4 °C for 10 min before the loading on column HR 10/30, pre-balanced with a 20 mM Tris-HCl at pH8.0 & 150 mM NaCl. Furthermore, three-angle refractive index detectors for light dispersion were connected to the system and analyzed by the ASTRA software after every 0.5 s, providing a molar mass of each sample.

### 2.3 Native PAGE

Native PAGE using a fast method with 8–25% acrylamide gradient gels monitored the native state of POP3PYD (1–83 aa) and ASCPYD (1–96 aa), as well as the complex formation. Protein samples purified by chromatography were loaded on the gel, followed by bright blue coomassie stain.

### 2.4 Protein complex assay by size-exclusion chromatography

Purified protein samples of POP3 PYD domain of corresponding amino acid 1–83 and ASC PYD domain of corresponding amino acid 1–96 aa were mixed in a molar ratio of approximately 1:1 and subjected to pre-incubation for 60 min at 4 °C. The protein mixture was further purified via superdex 200 gel filtration column HR 10/30 equilibrated by 20 mM Tris-HCl at pH 8.0 & 150mMNaCl.

## 3 Results

**Fig. 1:**
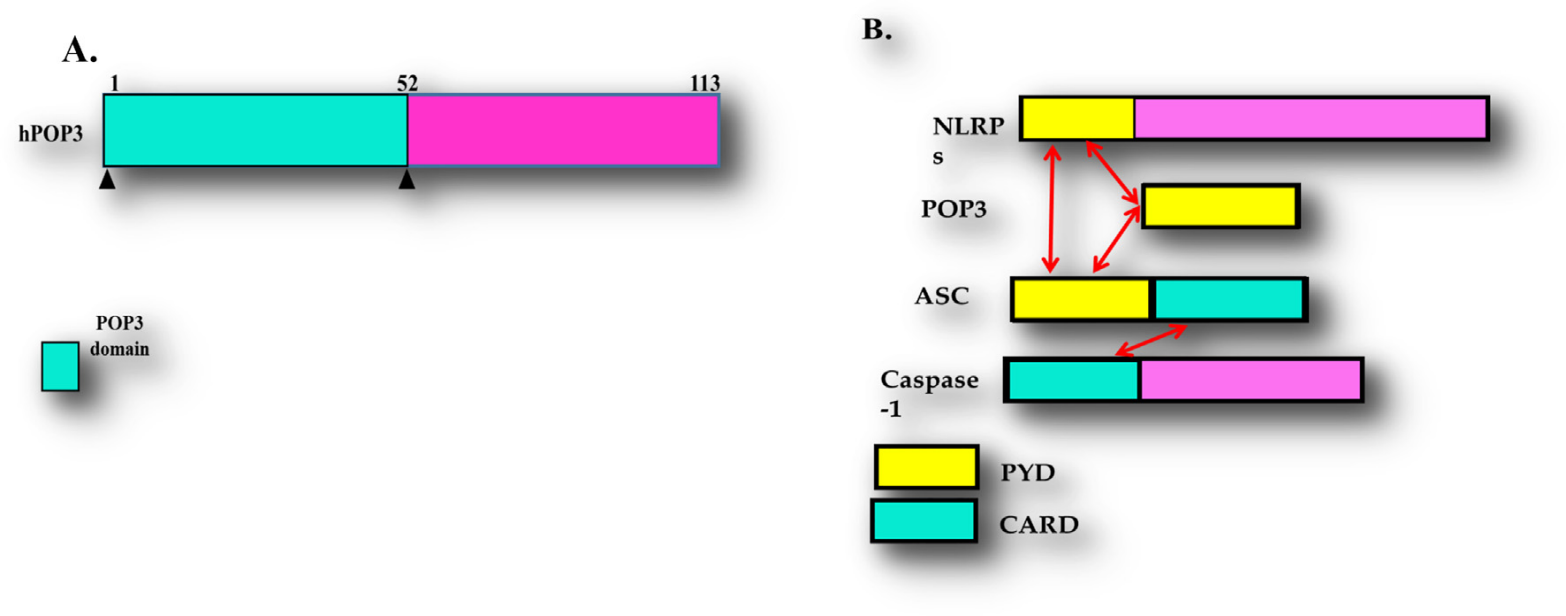
Schematic structure of Pyrin only protein 3 (POP3). (A) The domain boundary of PYD domain of POP3 with a number of amino acids from 1 to 83 aa. (B) Domain boundary of inflammasome proteins. Protein domain engaged in interactions are shown. Red arrows indicate particular interactions.

PYRIN Domain-Only Protein 3 (POP3) consists of 113 amino acids with PYD domain (Fig. 1A), which directly interacts with ASC PYD to block the binding of ASC to NLRPs (Fig. 1B). Four members of POPs have been identified, such as POP1, POP2, POP3, and POP4. POP1 shows 64% sequence identity with the PYD domain of ASC protein. Several in vivo studies show that POP3 binds to the ASC (Schroder & Tschopp, 2010).

**Fig. 2:**
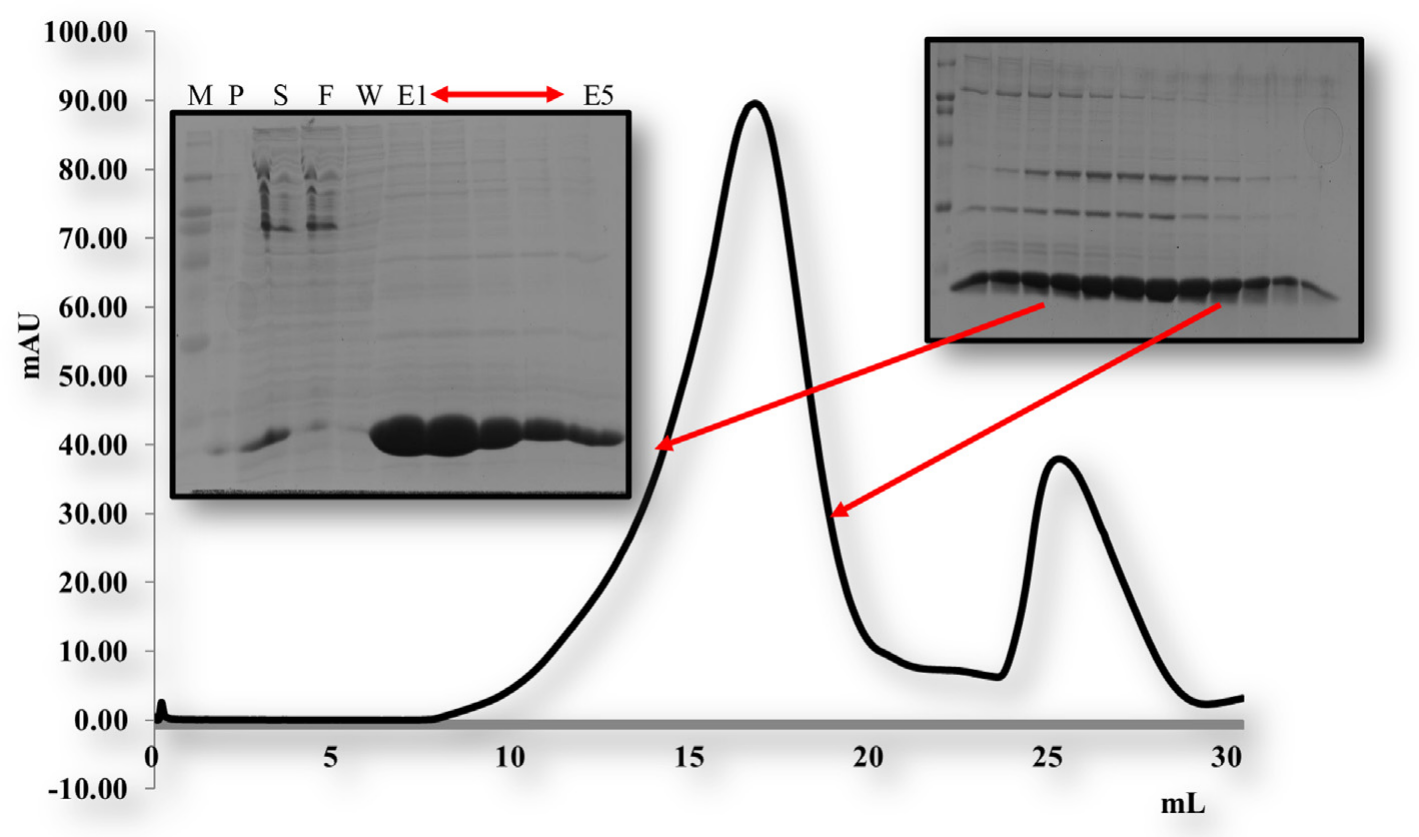
Gel filtration chromatogram. His tag and gel filtration chromatography of POP3 PYD (1–83 aa) domain in a 20 mM Tris (pH 8) 150 mM NaCl with SDS-PAGE shows both Ni-chromatography and purified fractions of gel filtration chromatography. The M# marker, P# pellet, S# supernatant, F# flow through, W# wash and E1-E5 (Elution).

For *in vitro* biochemical characterization, we produced the overexpression construct of each protein *viz*, POP3PYD (1–83 aa) and ASCPYD (1–96 aa). Each protein was over-expressed in bacterial cell BL21 and purified by Nickel chromatography and size exclusion chromatography (SEC). The SEC of, the POP3PYD domain with corresponding amino acid 1–83 aa, was eluted at approximately 16 mL in Tris and NaCl buffer conditions (Fig. 2). The gel filtration chromatography of the POP3PYD domain at low pH and high salt (500 mM NaCl, pH 5.0) showed an unstable behavior, and protein was not happy in those buffer conditions (Fig. 3a, Fig. 3b). The gel filtration chromatography of the ASCPYD domain of corresponding amino acid 1–96 was eluted between 10 and 18 mL at 20 mM Tris 150 mM NaCl buffer condition that shows oligomerization peak mediated by PYD domain (Fig. 4). Fig. 2 shows that the corresponding POP3 amino acid PYD DOMAIN [(1–83) (His-tag gel lanes)] migrates in the gel to a position close to the marker with molecular weight (15 kDa) with a measured His-tag (HHHHHH) molecular weight of 10,878 Da. Interestingly, our chromatography analysis exhibited that POP3 PYD was soluble at both pH 8.0 and pH 5.0 and came out in the correct place through the gel-filtration column. (Fig. 3a, Fig. 3b); however, the behavior of the target protein was not well at a pH of 5.0. After evaluating the effect of salt on the solubility of POP3 PYD domain (Figs. 2 and 3A). The POP3 monomeric PYD DOMAIN's measured molecular weight was 10,878 Da, and the experimental molecular weight in MALS was 19,000 Da, with a polydispersity of 1.04 for the POP3 PYD DOMAIN (Fig. 5). The results found on SEC and MALS, PYD DOMAIN of POP3 is a dimer in solution.

**Fig. 3a:**
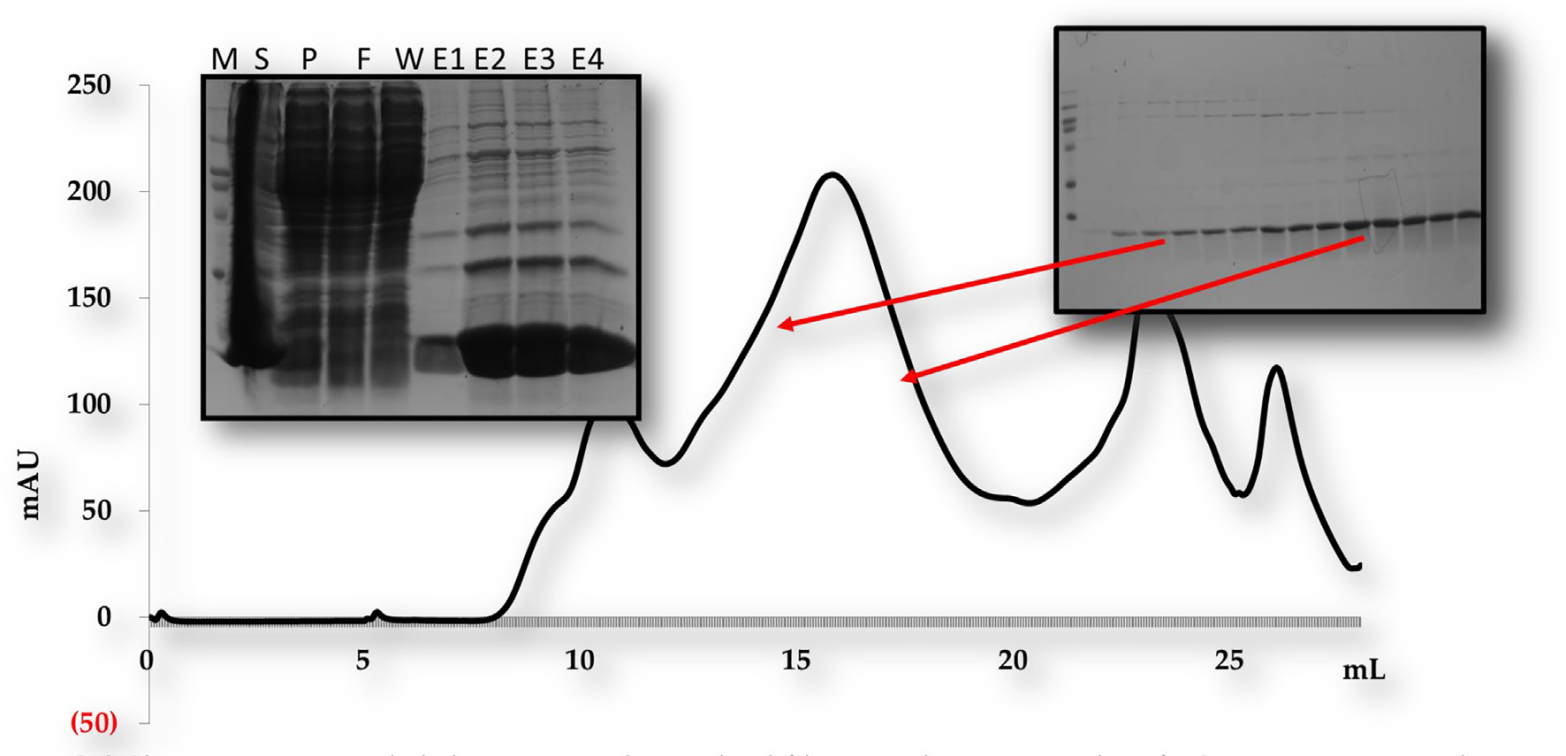
Gel Chromatogram. Nickel chromatography and gel filtration chromatography of POP3 PYD (1–83 aa) domain in a 20 mM Tris (pH 8) 500 mM NaCl with SDS-PAGE shows both Ni- chromatography and purified fractions of gel filtration chromatography. The M# marker, S# supernatant, P# pellet, F# flow through, W# wash and E1-E4 (Elution).

**Fig. 3b:**
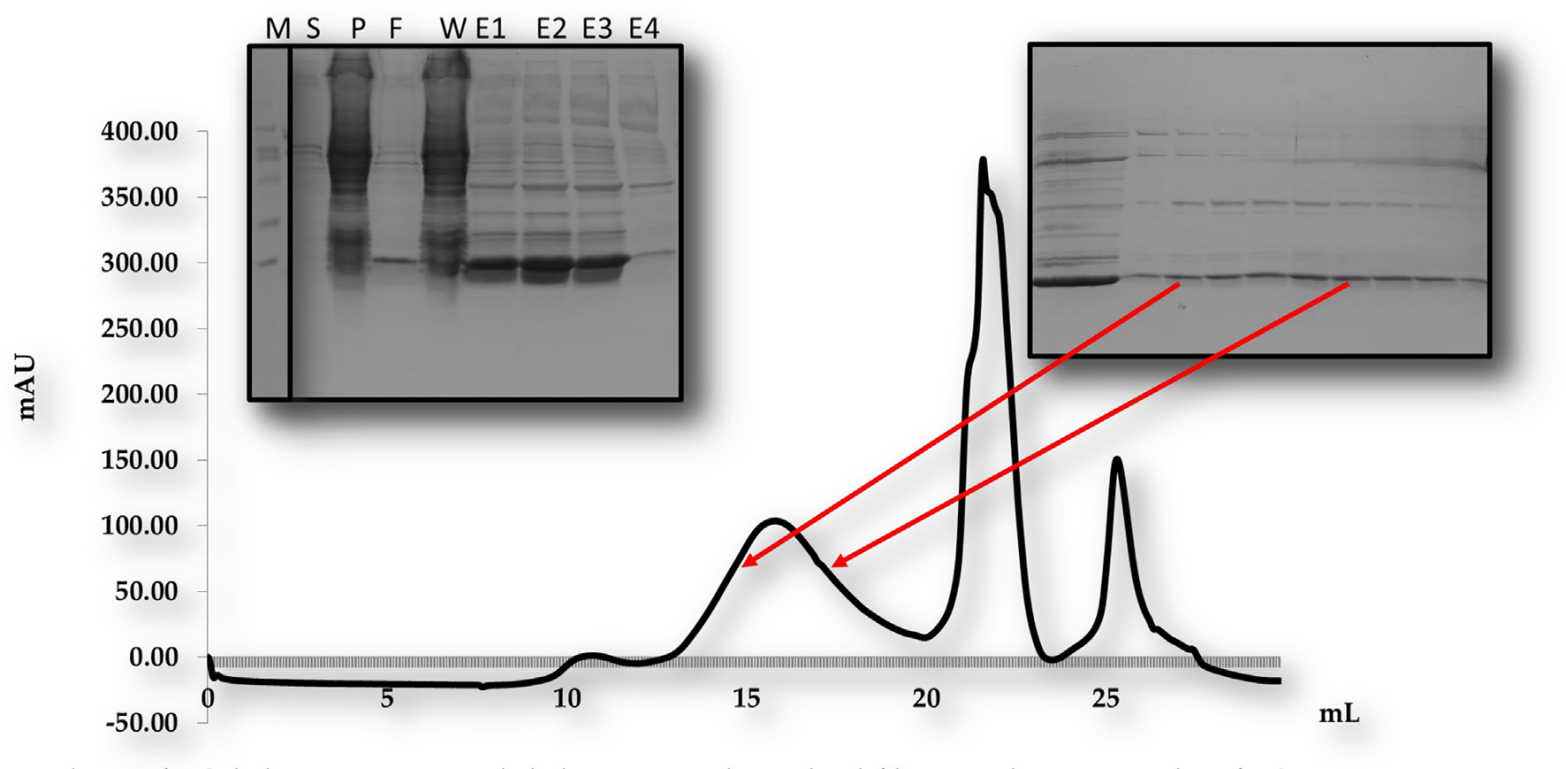
Gel chromatogram. Nickel chromatography and gel filtration chromatography of POP3 PYD (1–83 aa) domain in a 20 mM Sodium citrate (pH 5.0), 150 mM NaCl, 5 mM DTT with SDS-PAGE shows both Nickel-chromatography and purified fractions of gel filtration chromatography. The M# marker, S# supernatant, P# pellet, F# flow through, W# wash and E1-E4 (Elution).

**Fig. 4:**
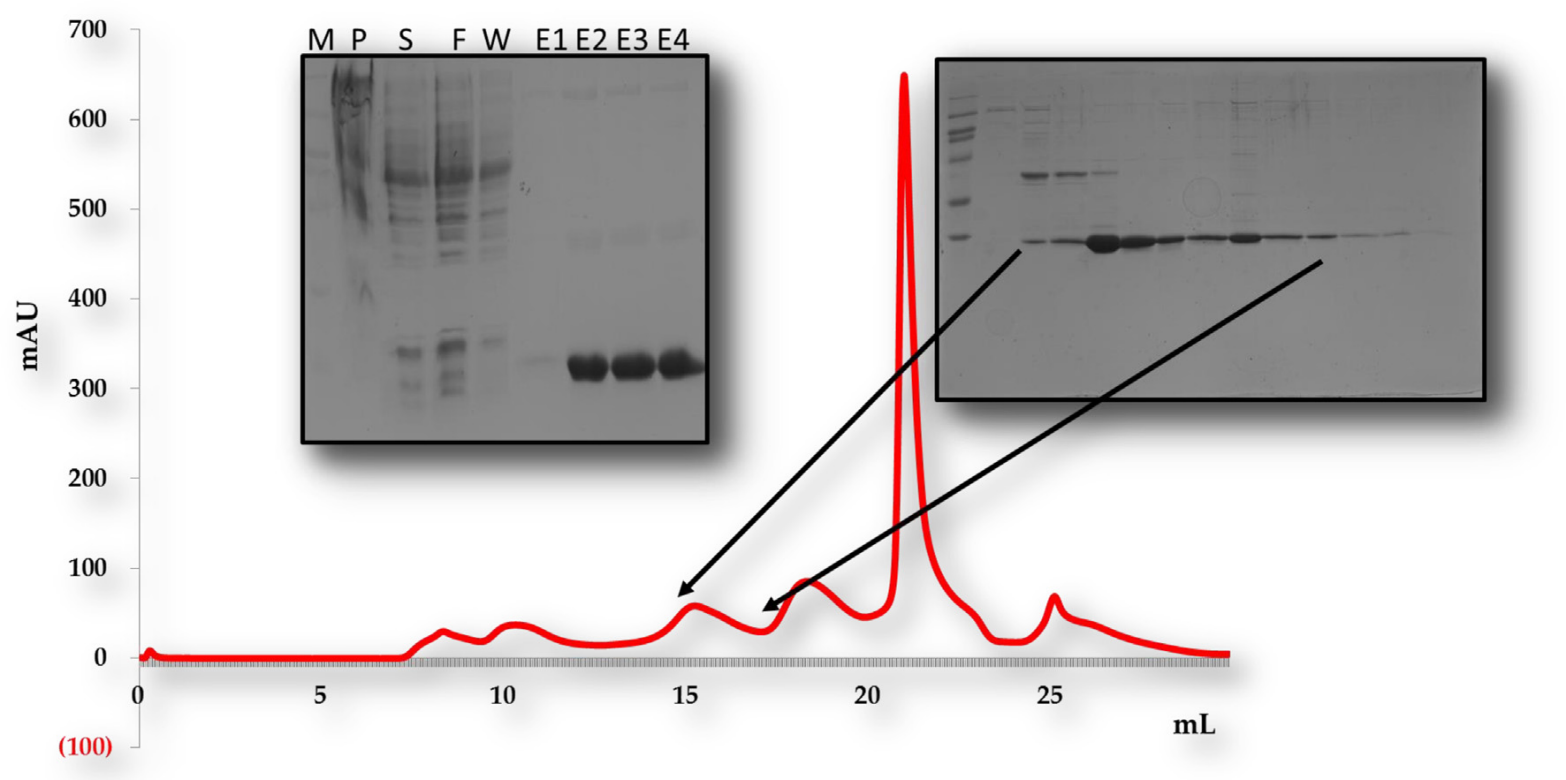
Gel chromatogram. Nickel chromatography and gel filtration chromatography of ASC PYD domain in a 20 mM Tris (pH 8) 150 mM NaCl with SDS-PAGE shows both Nickel chromatography and purified fractions of gel filtration chromatography. The M# marker, P# pellet, S# supernatant, F# flow through, W# wash and E1-E4 (Elution).

**Fig. 5:**
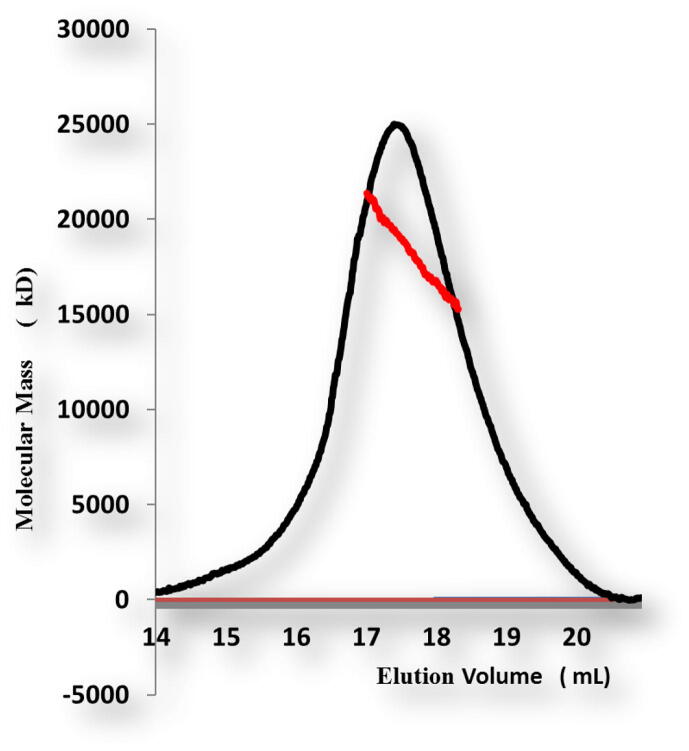
MALS result of POP3 PYD domain of corresponding amino acid 1–83 with 0.6% fitting error.

**Fig. 6:**
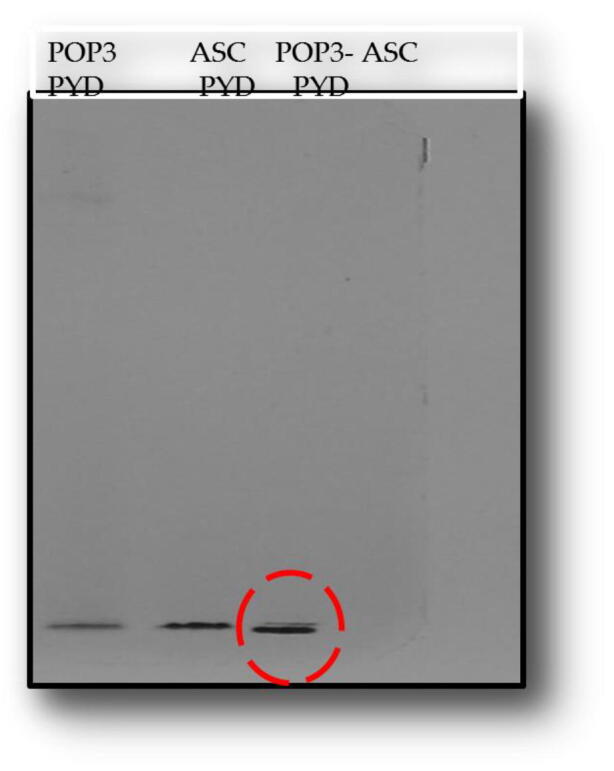
POP3PYD domain failed to interact with ASCPYD domain *in vitro specifically*. Native PAGE assay shift with construct of POP3PYD and ASCPYD. The red-dotted circle indicates no interaction not complex formation.

To investigate the complex assembly of PYD DOMAIN of POP3 (1–83 aa) and ASCPYD domain (1–96 aa) using structural and biochemical assays *in vitro*, different constructs were designed of PYD DOMAIN of POP3 shows in Table 1, known to intermingle with the ASC PYD (1–96 aa). Similarly, the ASC PYD domain, as shown in Table 2, was also designed constructs of interest to find the best overexpressed. The gel chromatography profile revealed that the PYD DOMAIN of the POP3 (1–83 aa) domain eluted at 16 mL, indicating a dimer's formation. The protein samples of purified PYD DOMAIN of POP3 (1–83 aa) and ASC PYD domain (1–96 aa) were used to perform native PAGE to confirm the formation of protein complex band. There was no association of either PYD DOMAIN of POP3 with the ASCPYD domain (Fig. 6). These results strongly indicate that the PYD DOMAIN of POP3 and ASCPYD domain has seen no interaction *in vitro*.

**Table 1:** Different constructs of Pyrin only protein 3 domain.

**Name**	**Species**	**Region**	**Enzyme**	**Plasmid**	**PCR**	**Cloning**	**Expression**
POP3-PYD-1	Human	1–83	NdeI/XhoI	pET24a	Done	Done	Over-expressed
POP3-PYD-2	Human	1–92	NdeI / XhoI	pET24a	Done	Done	Expressed
POP3-PYD-3	Human	1–104	NdeI / XhoI	pET24a	Done	Done	Expressed

**Table 2:** Different constructs of ASC PYD domain.

**Name**	**Species**	**Region**	**Enzyme**	**Plasmid**	**PCR**	**Cloning**	**Expression**
ASC-PYD-1	Human	1–91	NdeI/XhoI	pET24a	Done	Done	No expression
ASC-PYD-2	Human	1–96	NdeI / XhoI	pET24a	Done	Done	Over- Expression

**Fig. 7:**
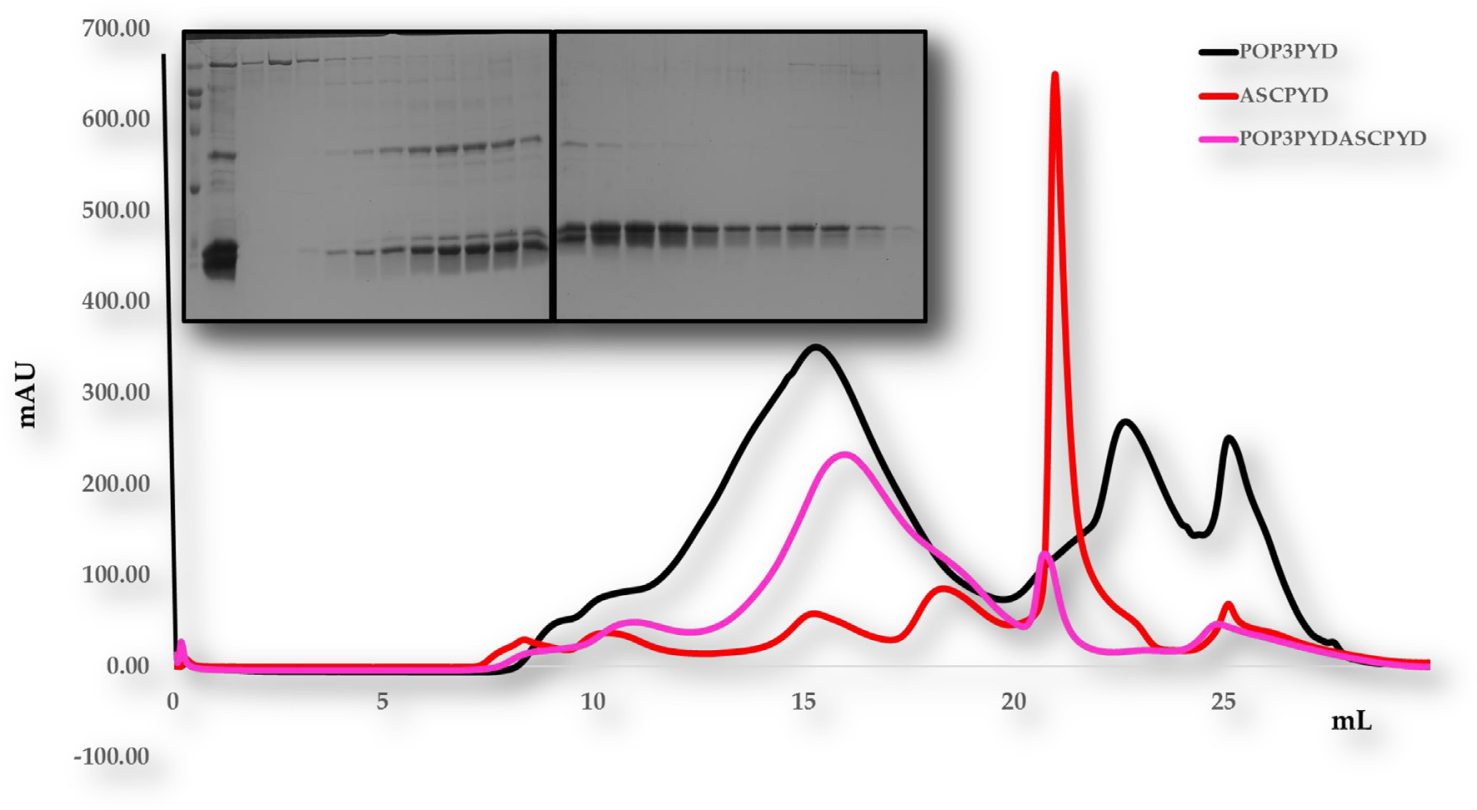
Gel filtration chromatogram. Size-exclusion chromatography (SEC) profiles of POP3 PYD domain shows no interaction with specifically ASC PYD domain *in vitro*. SDS-PAGE is shown on top right side of peak.

We performed size exclusion chromatography to confirm our results. The mixtures of PYD DOMAIN of POP3 (1–83 aa) did not produce complex peaks (Fig. 7). This result showed consistency with our previous native PAGE results, revealing that PYD DOMAIN of POP3 (1–83 aa) specifically did not associate with ASCPYD domain (1–96 aa) *in vitro* because of oligomerization of PYD domain inhibited assembly by self. Further research to understand the molecular function in vivo would give more insight into the PYD domain of POP3 and ASC PYD biology.

## 4 Discussion

Large molecular complexes are the formation of inflammasomes triggered by pathogens that guide to caspase-1 activation and are involved in the both inflammatory and adaptive immune response. The key components of inflammasome NALP3 are ASC, NALP3, and caspase-1, coupled with NOD-like receptors (NLRs). Each protein comprises the superfamily of the DD, caspase-1 CARD, ASC, and NALP3 PYD to permit protein module interaction. ASC constituted of both CARD and PYD domain at N-and C terminal, respectively. Interaction of ASCPYD and POP3 PYD is critical for inflammasome recruitment of other essential proteins for its activation. The structure of ASC PYD showed a bundle of 6– helix of the DD superfamily. However, no structure of the POP3PYD domain is reported to date, and also no *in vitro* analysis explaining the complex formation by PYD-PYD interaction.

To understand the biochemical insight on a molecular basis, the Human PYD domain of Pyrin only protein 3 and ASC PYD domain was used to elucidate its biochemical characterization together with *in vitro* complex assembly formation. Both proteins were purified through -affinity chromatography and size exclusion chromatography (SEC). The PYD domain of POP3 forms the dimer in solution based on our size exclusion chromatography results. The homogeneity of protein was detected on SDS PAGE gel. To confirm the stoichiometry, we performed the MALS of the PYD domain of POP3 protein. In addition, protein behavior was influenced by extremely low pH (pH5.0) conditions and high salt conditions (500 mM NaCl) and aggregated as detected on SDS PAGE gel. Based on our results, our *in vitro* study confirmed that self-oligomerization of ASC PYD domain inhibited assembly of the complex with PYD domain of pyrin only proteins 3 consistent with a previous study done by Narayanan KB et al. The homogeneity of this complex assembly was confirmed by SDS-PAGE.

**Fig. 8:**
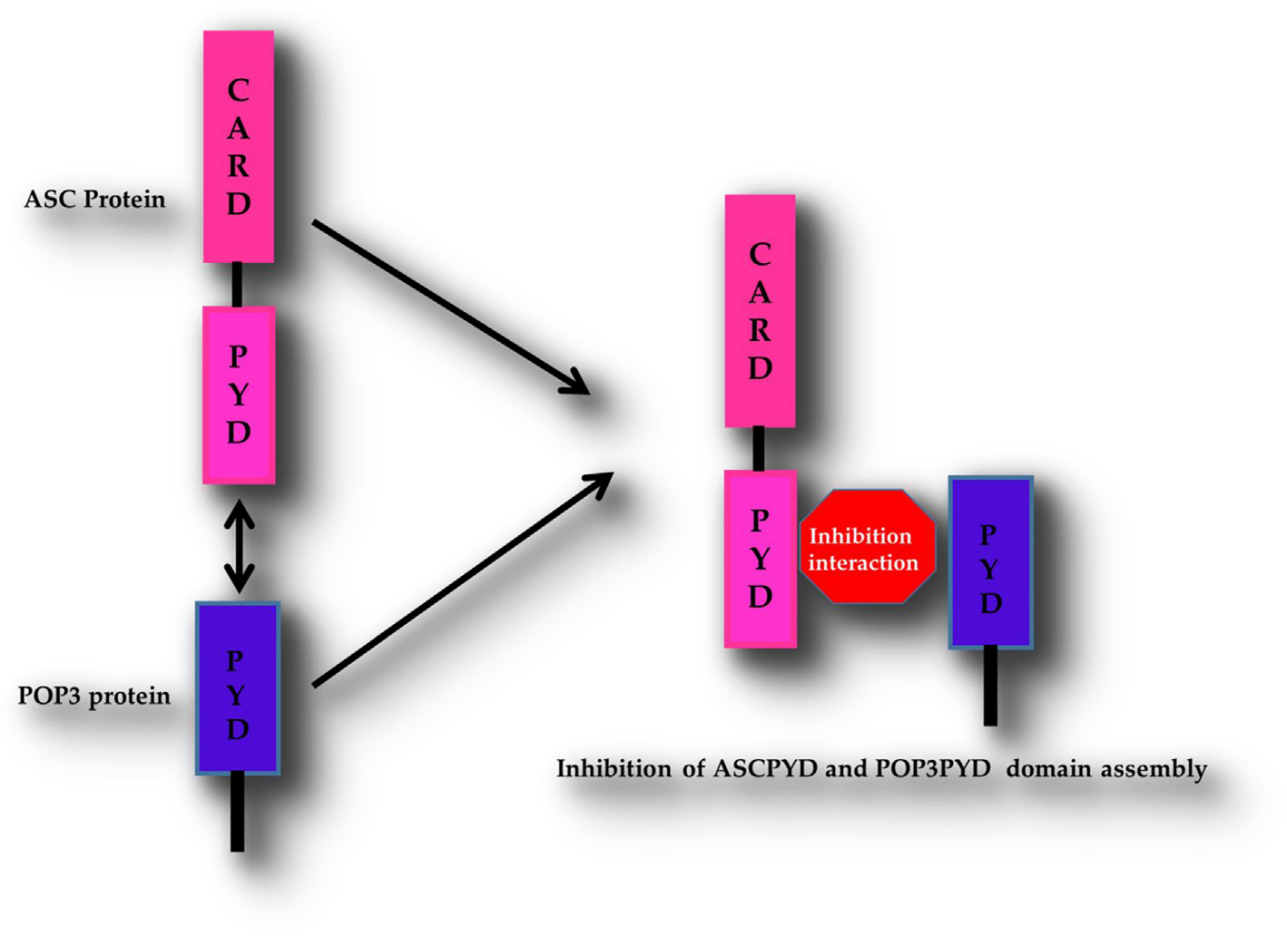
Potential *in vitro* inhibitory assembly complex by ASC PYD domain against POP3 PYD DOMAIN.

No specific interaction between the PYD domain of POP3 and ASC PYD domain was observed *in vitro*, and a particular binding region of POP3 PYD was analyzed. The PYD domain of ASC contributed to self-oligomerization, which inhibited the binding to the PYD domain of POP3 protein. This strategy may be necessary for the ASC PYD domain's inhibitory activity to the PYD domain of POP3 (Fig. 8).

## 5 Conclusion

Our study strongly demonstrated that the ASC PYD domain of corresponding amino acid 1-96aa easily self-oligomerized and prevented complex assembly with POP3 PYD (1–83 aa). Furthermore, we showed dimer formation of POP3 PYD in solution. Moreover, we find unstable POP3 PYD protein behavior in extremely low pH (pH5.0) conditions, and the proteins get aggregated at high salt conditions (500 mM NaCl). This can act as a template for further study to understand the molecular mechanisms, which will widen the field of pyrin-only protein biology.

## Declaration of Competing Interest

All authors declare that there are no conflicts of interest.
